# Clinical and socio-demographic determinants of pentazocine misuse among patients with sickle cell disease, Benin City, Nigeria: a case-control study

**DOI:** 10.11604/pamj.2019.34.88.17257

**Published:** 2019-10-14

**Authors:** Ademola Adewoyin, Oluwafemi Adeyemi, Nosimot Davies, Matilda Ojo

**Affiliations:** 1 Department of Haematology and Blood Transfusion, College of Medicine University of Lagos, Lagos, Nigeria; 2 Department of Haematology and Blood Transfusion, University of Benin Teaching Hospital, Benin City, Nigeria; 3 Department of Haematology and Blood Transfusion, Lagos University Teaching Hospital, Lagos, Nigeria

**Keywords:** Pentazocine, abuse, misuse, addiction, sickle cell disease, sickle cell anaemia, sosegon, opioid abuse, Nigeria

## Abstract

**Introduction:**

Opioids are a mainstay in sickle cell disease (SCD) pain care. Opioids are known to cause physical and/or psychological dependence. Increasingly, a significant number of Nigerian SCD patients ("Pentaholics") are observed to abuse pentazocine. This trend is associated with new patterns of medical complications. This study aimed to describe the local spectrum of pentazocine abuse complications and identify possible clinical and socio-demographic determinants.

**Methods:**

We conducted a case control (age matched) study involving 50 booked SCD patients (25 cases and 25 controls) receiving care at the University of Benin Teaching Hospital, Benin City, Nigeria. Relevant clinical and socio-demographic details were collected and analyzed. Associations of categorical variables were tested using chi square or Fishers exact test.

**Results:**

The median participants’ age and duration of pentazocine abuse/self-use were 32 and 7 years respectively. Pentazocine injections were gotten from local pharmacies and patent medicine stores without any need for physician prescriptions (84% of cases). The buttocks, the thigh and the upper arm/deltoid were the commonest site of injection. Major complications observed were chronic ulcers on the thigh, deep wounds with abscess, healed scar at multiple sites, lower limb swelling and venous thrombosis. Working in healthcare fields/hospitals (Doctor, Nurses, Pharmacists) was significantly associated with pentazocine abuse.

**Conclusion:**

Health personnel or hospital workers living with SCD are more likely to abuse pentazocine. There is need for prompt triage and optimal control of acute sickle pain, institutional protocols for pain management and strict regulations on supply of prescription drugs such as pentazocine.

## Introduction

Sickle cell disease (SCD) is a major public health challenge in Nigeria and many other sub-Sahara African nations [1-3]. SCD results from an inherited haemoglobin defect characterized by intra-corpuscular polymerization of haemoglobin molecules, which ultimately deforms the red cell into a crescent (sickle) shape [4, 5]. These abnormally shaped red cells undergo accelerated haemolysis, abnormal rheology with micro-vascular occlusion in various organ beds. Phenotypically, affected persons present with numerous complications affecting virtually every organ system. Acute complications include painful crisis, visceral sequestration crisis, hyperhaemolytic crisis, worsening (acute) anaemia, sickle priapism, acute chest syndrome, ischaemic stroke. Long term complications are age related and include sickle cell nephropathy, chronic liver disease, leg ulcers, ophthalmopathy, dilated cardiomyopathy, pulmonary hypertension, immunologic complications, as well psychosocial problems [4, 5]. Therefore, the goal of treatment is to modify the disease, prevent complications and treat existing complications/organ damage. However, SCD control in the Nigerian state is still largely suboptimal due to her huge population, high carrier prevalence, poor public health awareness, low government/political concerns, absence of neonatal detection schemes and dedicated sickle cell centers [6-8]. Delayed diagnosis among affected persons, low health infrastructure, ignorance and poverty all contribute to poor outcomes in SCD care in Nigeria [5-8]. Worst still, an increasing pattern of opioid abuse, self-inflicted/treatment related complication is being observed among affected persons [9-11]. Opioid are mainstay in SCD pain management. However, opioids may be associated with psychologic and or physical dependence. In Nigeria, pentazocine is the most readily available opioid analgesia for moderate to severe pain. Anecdoctal survey suggests that asides pentazocine and morphine (less available), other parenteral opioids are relatively unavailable, thus narrowing therapeutic options in the Nigerian setting. It is however undesirable that pentazocine, a synthetic opioid is associated with a new pattern of treatment related, self-inflicted medical complication [9-11]. As opined in a case series by Kotila *et al.* in Ibadan, opioid abuse is an emerging/understudied problem among SCD patients in Nigeria [9]. Similarly, Iheanacho *et al.* reported 11 SCD patients in Benin City, with history of chronic pentazocine abuse and physical complications including ulcers, scars, lymphedema, fibrous myopathies [10]. Mudrick C *et al.* also reported a Nigerian patient presenting in the US with necrotizing soft tissue infection and florid osteomyelitis resulting from injectable pentazocine use [11]. There is therefore a need to further characterize pentazocine abuse/misuse among affected persons living with sickle cell disease in Nigeria, in order to drive positive actions to mitigate this potential chronic menace. The objective of the study was to describe patterns of pentazocine abuse, associated medical complications as well as to identify possible clinical and socio-demographic determinants in a cohort of persons living with SCD, with a bid to proffering feasible solutions.

## Methods

The study was conducted among patients receiving care at the Adult Haematology Unit of the University of Benin Teaching Hospital, a tertiary health facility in Benin City, South-South Nigeria. This study was designed as a case control study involving 50 SCD participants. Cases included 25 known SCD patients with established history of pentazocine abuse or addiction. All cases were defined based on meeting 2 of 11 criteria (Table 1) within a year of diagnosis [12]. Control were SCD patients without documented clinical history or evidence of pentazocine addiction at the time of the study. Participants were recruited during outpatient consultations or ward admissions, after detailed explanation of the study protocol and informed consent obtained. Relevant data including socio-demographic details, haemoglobin phenotype and details regarding pentazocine abuse (duration and frequency of use, sites of injections, needle sharing, notable physical complications of abuse) were obtained using a structured, interviewer administered questionnaire. Questionnaire was pre-tested among 5 persons with SCD for construct and content validity, who were excluded from the main study. Data were logged and analyzed using the Statistical Package for Social Sciences (SPSS), version 16, Chicago USA. Frequencies and percentages were calculated for categorical data, with medians and interquartile range calculated for numerical data. Associations of categorical variables were tested using chi square or Fishers exact test. P values < 0.05 were considered significant.

**Table 1:** Substance abuse disorder, international statistical classification of disease and related health problems, ICD -10

Sub-groups	CRITERIA
**IMPAIRED CONTROL**	Consumption of greater amounts of the substance than intended Failed attempts to cut down use or abstain from the substance Increased amount of time spent acquiring, using or recovering from effects craving
**SOCIAL IMPAIRMENT**	Failure to fulfill responsibilities at work, school or home Continued substance use despite recurrent social or interpersonal problems secondary to the effects of such use (e.g. frequent arguments with spouse over the substance use) Isolation from life activities
**RISKY USE**	Use of substances in physically hazardous situations (e.g. driving while intoxicated) Continued substance abuse despite recurrent physical or psychological problems secondary to the effects of the substance use
**PHARMACOLOGIC**	Tolerance and use of progressively la rger amounts to obtain the same desired effect Withdrawal symptoms when not taking the substance

NB: tolerance and withdrawal are not needed to make diagnosis

## Results

The median age of study participants was 32 years (Table 2). Most had tertiary levels of education. About 16 (32%) studied or worked in a healthcare related field or industry. The predominant haemoglobin phenotype in the cohort was Haemoglobin SS (sickle cell anaemia). Regarding their first knowledge of pentazocine among cases, most of them (60%) discovered the drug during acute admissions for sickle crisis, another fraction (20%) learnt about pentazocine in the course of their medical/paramedical training. On the average, 36% and 20% of the affected persons take at least 2 amp (60mg) of pentazocine on daily or at most weekly basis in the last 7 years (Table 3). Some report to take as much as 30 amps (900mg) in a single bout/binge (Table 4). Most of them (84%) easily secure purchase of the drug from pharmacy outlets without a need for doctor's prescription. Others when required to provide prescription forms admit raising prescriptions for themselves. Commonest sites for self-injection of pentazocine were buttocks, thigh and upper arm/deltoid area (Table 3). No report of needle sharing was observed in index study. When asked about having to increase dose with time, 44% acknowledges some dose escalation to achieve same effect (Table 4). Some of the effects/complications of pentazocine misuse noted includes chronic ulcers, healed scars (not ankle ulcer or scars), abscess and deep wounds, woody legs/indurations, lymphedema, ankylosis, vascular injury and less commonly venous thrombosis (Table 4). No similar complication was observed in the control group. Reasons given for self-indulgence in pentazocine misuse included inconvenience of regular hospital care, faster pain relief than going to hospital, suboptimal pain control in the hospital, economic reasons (less cost compared to hospital care), as well as psychologic relief (Table 5). A significant proportion, 16 (64%) affected persons had previously failed in their attempts to discontinue pentazocine abuse or even medical rehabilitations (Table 5). When tested, participants who studied or worked in a healthcare related field were found to be significantly associated with pentazocine abuse with an odd of 4.85 (Table 6). Those with frequent painful crisis also showed a significant tendency to indulge in pentazocine misuse (odds of 4.47). Pentazocine abuse was significantly associated with additional physical morbidities in sickle cell disease among index participants (Table 6).

**Table 2:** Bio-data of the study participants N = 50 (100%)

Variables		Frequency (n)	Percentage (%)
**Age (years)**			
	15 – 34	36	72
	35 – 54	13	26
	55 and above	1	2
**Median = 32, Min = 16, Max = 61**			
**Sex**			
	Male	25	50
	Female	25	50
**Education**			
	Primary	-	-
	Secondary	5	10
	Tertiary	45	90
**Field of Work/Study**			
	Healthcare related	16	32
	Law/Legal	2	4
	Arts	1	2
	Business/ management	11	22
	Engineering	2	4
	Education	4	8
	Artisan/Apprentice	2	4
	Physical/life science	4	8
	Agriculture	1	2
	Social sciences	3	6
	None	4	8
**Employment status**			
	Student	17	34
	Employed	21	42
	Unemployed	11	22
	Retired	1	2
**Haemoglobin phenotype**			
	SS	46	92
	SC	4	8

N = 50 (100%)

**Table 3:** Details of pentazocine abuse among cases

Variables	Frequency (n)	Percentage (%)
**First knowledge**		
During hospital admission for sickle crisis	15	60
As an health worker/student	5	20
Injection treatments from pharmacy/chemist shops	1	4
Told/introduced to self-use by an SCD friend	1	4
During home based treatment by nurses	3	12
**Duration of self pentazocine use (years)**		
1 year or less	3	12
2 – 5	8	32
6 or more	14	56
**Median duration of use = 7**		
**Frequency of pentazocine use**		
Everyday	9	36
At least 1/week	5	20
At least 1/month	7	28
At least 1/ 3 month	2	8
Only in painful crisis	2	8
**Procurement of pentazocine**		
Pharmacy outlets	18	72
Chemist/Patent medicine	7	28
**Need for prescription**		
Yes	4*	16
No	21	84
Body sites for Pentazocine injections**		
Buttocks	20	80
Thigh muscle	18	72
Intravenous	1	4
Upper arm/Deltoid	10	40
Calf/Leg	1	4
Popliteal area	1	4
Breast	1	4
**Needle sharing**		
Yes	-	-
No	25	100

N = 25 (100%)

* self prescribe it

** multiple responses

**Table 4:** Practices regarding pentazocine self-use

Variables	Frequency (n)	Percentage (%)
Use when not in physical pains		
Yes	12	48
No	13	52
DOSE increment with time		
Yes	11	44
No	14	56
Regular/usual dose per time (Ampoule*)		
1	11	44
2	11	44
3	3	12
Mean±SEM = 1.68±0.14, Median = 2, Min = 1, Max = 3		
Maximum dose per time (ampoule)		
1-2	16	64
3-5	6	24
6 or more	3	12
Mean±SEM = 3.84±1.18, Median = 2, Min = 1, Max = 30		
Effects/Complications of Pentazocine use		
Chronic Ulcers	12	48
Healed Scars	11	44
Induration/woodiness	5	20
Abscess/Wound	10	40
Sedation	4	16
Euphoria	3	12
Leg Swelling	4	16
Ankylosis	2	8
Fainting episodes	2	8
Nausea/vomiting	1	4
DVT	1	4
Seizures	2	8
Vascular injury	1	4

N = 25 (100%), 1 ampoule= 30mg, DVT = deep venous thrombosis

**Table 5:** Reasons for pentazocine abuse

Variables	Frequency(n)	Percentage (%)
**Why prefer self-administration of pentazocine?**		
Not for pain. For psychologic relief	1	4
Don’t want to be admitted in the hospital	1	4
Does not get as much as he wants in the hospital	1	4
It’s is more convenient than hospital care	3	12
Faster pain relief than going to hospital	1	1
Distance to hospital especially at night	4	16
Cheaper than going to the hospital	3	12
Delay in prompt pain relief in hospital/suboptimal relief	6	24
Delay in being taken to hospital by relatives	2	8
**In the event of painful crisis, do you get prompt pain relief at the health facility?**		
Yes	17	68
No	8	32
**Have you had any previous/failed attempts to discontinue pentazocine abuse or any form of drug rehabilitation?**		
Yes	16	64
No	9	36

**Table 6:** Clinical and socio-demographic associations of pentazocine abuse

Variables	Frequency(n)			Test statistic
	cases	control	total	
**Sex**				X^2^=0.720, p = 0.396
Male	14	11	25	
Female	11	14	25	
**Education**				p = 0.500
Secondary	2	3	5	
Tertiary	23	22	45	
**Field of work/study**			X^2^=5.882, p = 0.015, OR = 4.85	
Healthcare related	12	4	16	
Non health related	13	21	34	
**Employment status**				X^2^=1.821, p = 0.402
Student	8	9	17	
Employed	9	12	21	
Unemployed	7	4	11	
Retired	1	0	1	
**Haemoglobin phenotype**				p = 0.055, AOR = 10.67
SS	25	21	46	
SC	0	4	4	
**Frequency of bone pains**				p = 0.069, OR=4.47
At least 1/month	7	2	9	
Less than 1/month	18	23	41	
**Physical complications**			p = 0.000, AOR = 125.80	
Yes	18	0	18	
No	7	25	32	

N = 50, OR = Odds ratio, AOR = Adjusted Odds Ratio

## Discussion

Pentazocine is a main-stay opioid in acute care of moderate-severe painful crisis in Nigerian SCD. Though highly desirable due to limited, locally available opioid choices, pentazocine is associated with use disorders/addictions, with myriads of physical complications, as reported in index study. Similar to other studies, most affected SCD persons were in their second to fourth decades [10, 13]. This is particularly appalling as young vibrant persons embattled with a background sickle cell disease in a sub optimal care setting are further incapacitated by addictions, leading to reduced economic productivity, poor work/educational output and increased healthcare costs. This study attempted to identify key clinical and or socio-demographic determinants of pentazocine abuse. Most of these subjects had their first exposure to pentazocine while being managed at home or facility for acute pain. It is interesting to note that SCD cases with pentazocine abuse were more likely engaged in healthcare-related job roles, compared to controls. The odds of pentazocine abuse is about five-fold in SCD persons working in healthcare related fields as doctors, pharmacists, etc. Perhaps, working in health-related fields has encouraged easier access to pentazocine either directly from physicians raising misguided prescriptions or undue access to pharmacy services. Some of the cases admitted to signing prescription sheets themselves. Similar observations are reported in other studies [10, 13]. Pentazocine is classified as a controlled drug in countries such as the USA, where it is grouped under schedule IV in the controlled drug substance [14]. However, drug regulation especially for controlled drugs such as pentazocine is still at a low practice in Nigeria. The proliferation of patent medicine stores as well as pharmacy outlets without proper professional registration/regulations is perhaps contributory. Indiscriminate sales of prescription drugs without physician recommendation is a major culprit. Over 80% admit to procuring pentazocine from pharmacy outlets without requiring physician's prescriptions, irrespective of the quantity and frequency of demand. Some of these pharmacy attendants connive with them in these purchases, because there are no effective policies to check these ills and excesses. Relevant stakeholders, government or non-governmental, as well as relevant professional and regulatory bodies will need to rise to stem this negative trend [15].

**Figure 1:**
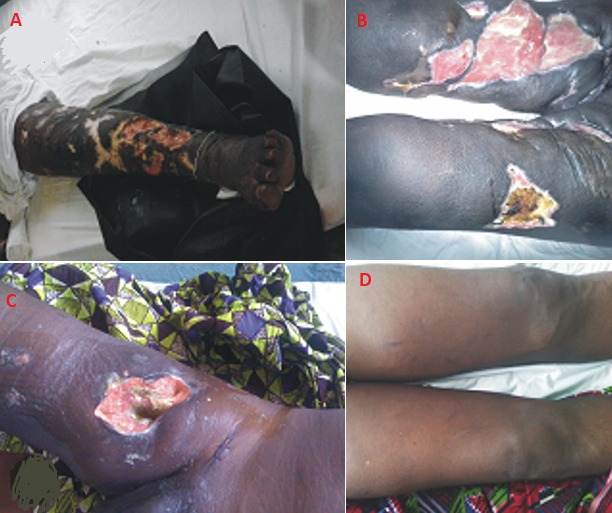
Physical complications of pentazocine abuse: (A,B) multiple coalescing chronic leg ulcerations with muscle involvements (separate cases); (C) vascular injury and bleeding from a thigh vessel, requiring surgical repair; (D) deep venous thrombosis of the right thigh (with sonologic evidence), coupled with bilateral ankyloses of the knees

Furthermore, it is being suggested that the tendency for self-medication and eventual degeneration to drug dependency/addiction may be heightened by inadequate acute pain care at health facilities or infusion rooms. Willful denial of copious opioid administration by care-givers due to fear of addiction or suspicion of malingering, negative health-seeking behavior by the affected persons, limited access to healthcare and poverty may all be contributory drivers to pentazocine use disorder. With the foregoing, pain control protocol must be instituted in dedicated care settings to achieve optimal control within 30 minutes of acute presentation, regular pain scoring for breakthrough pain and fixed dose analgesic schedules, coupled with individualized care based on prior patient needs, comorbidities, and other clinical guides. Patient controlled analgesia is also worth consideration. This should promote good health seeking attitudes, rather than self-indulgences [9, 16]. Notably, the commonest sites of self-injection were the buttocks and thigh muscles (Figure 1). These areas are concealed from family and friends and not likely to draw unsolicited attention until the complications become apparent. Fewer areas include the upper limbs and occasionally the trunk. The risk of needle sharing is far less when compared to intravenous drug abusers especially for illicit drugs. These patients do not seek the company of others as the initial purpose is to tackle pain and thereafter lapse into the addictive phase. They may still be potential for abuse of other illicit drugs and hence likely at risk for HIV/AIDS or hepatitis transmission. The most common complications observed in index study included chronic ulcers involving either the upper or lower limbs or both and abscess formation in soft tissues of the limbs. Cutaneous manifestations including dermal fibrosis, granulomatous inflammation, and vascular thrombosis are also well documented in the literature and also observed in index cases as in Figure 1 [17, 18]. Pentazocine injected intramuscularly into all four limbs and the trunk-in one case even the back-has produced symptoms suggesting the "stiff man syndrome", with hard, tender muscles and disabling contractures. Focal precipitation of acidic pentazocine is probably responsible.

## Conclusion

Pentazocine misuse among patients with SCD in our community does occur. Hospital workers, health personnel or trainees living with SCD are more likely to abuse pentazocine indiscriminately. This trend (*"pentaholism''*) calls for appropriate comprehensive SCD control program to mitigate further indulgences. The management of SCD predisposes families to physical, psychosocial and financial stress, which will be invariably worsened by drug dependence. This also underscores the need for prompt triage/optimal control of acute sickle pain, institutional protocols for pain management, and strict regulations on supply of controlled drugs such as pentazocine.

### What is known about this topic

- Sickle cell disease is associated with opioid abuse;
- Opioid dependence and over-dosage causes physical and psychological complications.

### What this study adds

- Healthcare workers or hospital personnel living with SCD are at a greater tendency to abuse Pentazocine.
